# Non-invasive optical spectroscopic monitoring of breast development during puberty

**DOI:** 10.1186/s13058-017-0805-x

**Published:** 2017-02-06

**Authors:** Lothar Lilge, Mary Beth Terry, Jane Walter, Dushanthi Pinnaduwage, Gord Glendon, Danielle Hanna, Mai-Liis Tammemagi, Angela Bradbury, Saundra Buys, Mary Daly, Esther M. John, Julia A. Knight, Irene L. Andrulis

**Affiliations:** 1 Princess Margaret Cancer Centre, University Health Network, 101 College Street, Toronto, ON M5G1L7 Canada; 2 Medical Biophysics, University of Toronto, 101 College Street, Toronto, ON M5G1L7 Canada; 3 Department of Epidemiology, Columbia University Mailman School of Public Health, New York, NY USA; 4 Herbert Irving Comprehensive Cancer Center, Columbia University Medical Center, New York, NY USA; 5 Lunenfeld-Tanenbaum Research Institute, Sinai Health System, 600 University Avenue, Toronto, ON M5G 1X5 Canada; 6 Departments of Medicine and Medical Ethics & Health Policy, Perelman School of Medicine of the University of Pennsylvania, Philadelphia, PA USA; 7 Department of Medicine, Huntsman Cancer Institute, University of Utah School of Medicine, Salt Lake City, UT USA; 8 Department of Clinical Genetics, Fox Chase Cancer Center, Philadelphia, PA USA; 9 Cancer Prevention Institute of California, Fremont, CA USA; 10 Department of Health Research & Policy (Epidemiology), and Stanford Cancer Institute, Stanford School of Medicine, Stanford, CA USA; 11 Division of Epidemiology, Dalla Lana School of Public Health, University of Toronto, Toronto, ON Canada; 12 Department of Molecular Genetics, University of Toronto, Toronto, ON Canada

**Keywords:** Optical spectroscopy, Breast development, Breast cancer family history, Tanner staging, LEGACY girls study

## Abstract

**Background:**

Tanner staging (TS), a five-stage classification indicating no breast tissue (TS1) to full breast development (TS5), is used both in health research and clinical care to assess the onset of breast development (TS2) and duration in each stage. Currently, TS is measured both visually and through palpation but non-invasive methods will improve comparisons across settings.

**Methods:**

We used optical spectroscopy (OS) measures from 102 girls at the Ontario site of the LEGACY girls study (average age 12 years, range 10.0–15.4 years) to determine whether breast tissue optical properties map to each TS. We further examined whether these properties differed by age, body mass index (BMI), and breast cancer risk score (BCRS) by examining the major principal components (PC).

**Results:**

Age and BMI increased linearly with increasing TS. Eight PCs explained 99.9% of the variation in OS data. Unlike the linear increase with age and BMI, OS components had distinct patterns by TS: the onset of breast development (TS1 to TS2) was marked by elevation of PC3 scores indicating an increase in adipose tissue and decrease in signal from the pectoral muscle; transition to TS3 was marked by elevation of PC6 and PC7 and decline of PC2 scores indicating an increase in glandular or dense tissue; and transition to TS4+ by decline of PC2 scores representing a further increase in glandular tissue relative to adipose tissue. Of the eight PCs, three component scores (PC4, PC5, and PC8) remained in the best-fitting model of BCRS, suggesting different levels of collagen in the breast tissue by BCRS.

**Conclusions:**

Our results suggest that serial measures of OS, a non-invasive assessment of breast tissue characteristics, can be used as an objective outcome that does not rely on visual inspection or palpation, for studying drivers of breast development.

**Electronic supplementary material:**

The online version of this article (doi:10.1186/s13058-017-0805-x) contains supplementary material, which is available to authorized users.

## Background

Breast cancer (BC) incidence is increasing in women under age 40 years in the US [1] and is increasingly common worldwide in women under age 50 years [2, 3]. Decline in the age of breast development [4] may account for some of the change. Age of menarche, a long-established risk factor for breast cancer, has been relatively stable in recent decades [5]. As the interval between early breast development and the age at menarche (referred to as pubertal tempo) when the breast may be more susceptible to carcinogens has widened, it is essential to have other measures of pubertal development [6]. Height, age at breast development, age at menarche, and increased tempo were each independently associated with an increase in BC risk in a large prospective cohort study [7]. Compared with height and age at menarche, age at breast development has been more challenging to determine.

Breast development is often assessed using Tanner stages (TS), which is routinely used in clinical evaluation. TS range from TS1 to TS5, and are separately evaluated for breast and pubic hair. We focus this paper on breast TS with TS1 referring to no breast development, TS2 as the first appearance of breast buds, TS3 where the areola and breast are larger than just buds but the areola does not stick out away from the breast, TS4 where the nipple is raised above the breast, and TS5 the mature breast. Tanner stage is generally assessed by a clinician using visual inspection followed with palpation, but can also be evaluated by self-reporting or maternal reporting using drawings of TS with explanatory text [8]. TS reporting by parents or self-reporting has been less reliable and valid compared with clinician reports, with parents more accurate reporters of TS in children before age 11 years and children more accurate reporters after age 11 [9].

Breast development can also be tracked through imaging methods, although most imaging methods such as dual-energy x-ray absorptiometry, magnetic resonance imaging or mammography are either too expensive to use routinely in young girls and/or involve exposing the breast to ionizing radiation. Breast tissue composition is associated with mammographic breast density (MBD), which represents the connective and glandular versus the adipose tissue fraction [10–12]. The tissue components giving rise to MBD have distinct optical absorption spectra, which led to the development of optical spectroscopy (OS) methods to examine breast tissue composition using visible and near infrared light. OS has been shown to identify women of mammographic screening age having >75% MBD [13] and who are at elevated risk of BC, with sensitivity and specificity >0.9 [14, 15]. Studies in younger women (31–40 years of age) showed strong associations with parity [16], another well-established BC risk factor. Here we present an extension of the OS technique adapted for the developing breast of girls ages ≥10 years, to demonstrate the utility of this method to detect breast development TS, adjusting for age, BMI, and breast cancer risk score (BCRS). We further examined whether BCRS was associated with OS components.

## Methods

### Study population

The participants in this study were from the Ontario site of the LEGACY girls study [17], an NCI-funded prospective cohort of 1040 girls enrolled at ages 6–13 years at five study sites in the US and Canada. Half of the girls come from families with positive BC history (BCFH+) defined as having at least one first or second degree relative diagnosed with BC. Girls without a breast cancer family history (BCFH-) had no first or second degree relative with BC. All participating institutions obtained Institutional Review Board approval (for more details see [17] and www.legacygirlstudy.org).

Of the girls from the Ontario site who were 10 years and older and who were invited to participate in the OS study, 93% accepted and completed baseline and follow-up measures. There were 105 Ontario girls initially eligible for this pilot study, with 102 complete datasets for analysis.

### OS instrumentation and data preparation for final analysis

The OS approach was similar to that previously described in adult women [18, 19] except for using light diffusely reflected from the tissue rather than transmitted through the breast, as TS1 to TS3 do not provide sufficient tissue to place the optical fiber bundles at opposite sides of the breast for transmission experiments [20, 21]. Reflectance quantification covered the 635–1060 nm spectral range. A 5-mm fiber bundle delivered broadband light from a halogen lamp to the skin surface and a 3-mm fiber bundle collected the diffuse reflected photons guiding it to the holographic transmission spectrophotometer (PPO, Kitchener, ON, Canada) with a cooled 256 × 1440 pixel CCD (Photometrics, NJ, USA). A black flexible template (shown in Additional file 1: Figure S1A), provided reproducible inter-optode distances and absorbed all photons reaching the surface. The participant was in the supine position and optical measurements were executed at four quadrants (Additional file 1: Figure S1B), superior, lateral, inferior, and medial on each breast, resulting in eight diffuse reflectance spectra per participant. The light source irradiance (approximately 180 mWcm^−2^) equals approximately twice the noontime solar exposure during the summer solstice in Boston, MA, USA, but does not contain UV or blue spectral components. Exposure times were 2–80 sec per spectrum.

Spectra were corrected for exposure time and dark signal, and a 7-point boxcar smoothing algorithm was applied followed by a cubic spline interpolation to sample spectra at 1 nm increments. Spectra were corrected for variations in the instrument throughput using a high albedo reflection standard, resulting in effective light attenuation spectra. Corrected spectra were mean-centered for principal component analysis (PCA). While two inter-optode distances (1.5 and 3 cm) were used, the short distance at times resulted in suspect detector saturation effects and was not further considered in this analysis; thus, 840 spectra were used to determine orthogonal PCA spectra reducing the dimensionality of information in each original spectrum. The eight first component vectors (PC1–PC8) (see Additional file 1: Figure S2) represent 99.99% of the variation seen in the complete dataset. Each principal component (PC) spectrum represents different optical tissue features, including light scattering by cellular and structural components, and absorption dominated by the five main breast tissue components (water, lipid, oxy-hemoglobin (HbO_2_), deoxy-hemoglobin (Hb), and collagen) and residual absorption by yet unidentified chromophores. As the breast develops homogenously bilaterally and only tissue average properties are sought, each PC score was averaged over both breasts resulting in an OS dataset comprising 105 girls, each having one score for each of the eight principal components (PC1–PC8).

#### Breast cancer risk score

We calculated a continuous probability score reflecting each girl’s estimated absolute lifetime risk of breast cancer. We estimated the BCRS based on available detailed pedigree data, allowing us to calculate a risk score using the breast and ovarian analysis of disease incidence and carrier estimation algorithm (BOADICEA) [22–24].

### Statistical methods

Complete data on age, body mass index (BMI) collected through clinical measures, Tanner breast stage assessed by a guardian, and BCRS were available for 102 of the 105 girls. Due to a small sample of girls in the TS5 group (*n* = 6) and the fact that some adolescent girls go directly from TS3 to TS5 without a TS4 or do not progress to TS5, we combined TS4 and TS5 for the analyses.

We used descriptive statistics to summarize the data. Analysis of variance (ANOVA) and univariate logistic regression were performed to identify covariates from PC1–PC8 scores, age, BMI and BCRS that predict breast stage. We also incorporated random forest analysis to examine the influence of all covariates together in the prediction, as multivariate logistic regression predictions are not reliable in a small dataset with many covariates. The features selected were used in multivariate logistic regression models. Linear discriminant analysis (LDA) with 60% of the data used as a training set and the rest used as a test set was applied to measure the predictive ability. We also examined the ability of OS measurements to predict BCRS. The best predictive model was selected by Akaike’s information criteria (AIC) [25]. Before conducting the above analyses, each PC score and BCRS were rescaled by dividing by the corresponding interquartile range for meaningful interpretation of the results. Correlation, regression, ANOVA and LDA analyses were performed using SAS 9.1 software (SAS Institute, Inc.) and the other analyses and plots were achieved using R statistical software, version 2.15.0 (http://www.r-project.org).

## Results

### Study cohort characteristics

Table 1 summarizes descriptive statistics for the cohort. The average age of the girls was 12.0 years with a range of 10.0–15.4 years. The average BMI was 18.6 with a range of 12.5–33.2. The average BCRS was 15% lifetime risk with a range of 11–30%.

**Table 1:** Characteristics of the cohort

Characteristic	Number	Percentage
Age (years)		
10 to <12	63	61.8
12 to <14	25	24.5
> = 14	14	13.7
Mean age = 12.03, SD = 1.43, minimum = 10.01, maximum = 15.38		
BMI		
10 to <15	6	5.9
15 to <20	71	69.6
20 to <25	19	18.6
25+	6	5.9
Mean BMI = 18.58, SD = 3.11, minimum = 12.46, maximum = 33.18		
Breast Tanner stage		
1	16	15.6
2	31	30.4
3	31	30.4
4	18	17.7
5	6	5.9
Breast cancer risk category		
BCRS <0.12	41	40.2
0.12 <= BCRS <0.2	46	45.1
BCRS > = 0.2	15	14.7
Mean BCRS = 0.15, SD = 0.05, minimum = 0.11, maximum = 0.30		

*BMI* body mass index, *BCRS* breast cancer risk score

### The association between OS measurements and breast TS

Eight PCs explained >99.9% of the variation in OS. Table 2, summary statistics (Additional file 2: Table S1A) and Additional file 1: Figure S3A show the association between age, BMI, and each OS PC with breast stage (TS1, TS2, TS3, and TS4). Breast stage increased with increasing age and BMI, as expected (*P* < 0.01). Unlike the linear increase with age and BMI, OS components had distinct patterns by TS: the onset of breast development (TS1 to TS2) was marked by elevation of PC3 scores; transition to TS3 was marked by elevation of PC6 and PC7 and decline of PC2 scores; and transition to TS4+ by the decline of PC2 scores.

**Table 2:** Association between breast Tanner stage and age, BMI, BCRS, and OS principal component scores

	Breast Tanner stage				
Predictor	1	2	3	4	
	Mean	Mean	Mean	Mean	*P* value
Age	11.05	11.19	12.32	13.58	<0.01
BMI	16.50	17.31	18.90	19.70	<0.01
BCRS	1.70	2.04	1.80	1.88	0.23
PC1	0.190	0.001	0.102	−0.368	0.26
PC2	0.33	0.31	−0.23	−0.41	<0.01
PC3	−0.62	0.11	0.15	0.10	<0.01
PC4	0..04	−0.04	−0.03	0.14	0.84
PC5	−0.07	−0.08	0.01	0.22	0.60
PC6	−0.34	−0.27	0.28	0.24	<0.01
PC7	−0.23	−0.20	0.20	0.24	0.06
PC8	−0.12	0.05	−0.01	0.25	0.60

*BMI* body mass index, *BCRS* breast cancer risk score, *PC* principal component, *OS* optical spectroscopy

Variable importance plots (Additional file 1: Figure S4A) by random forest feature selection confirmed some of these factors as important predictors in classifying girls into four breast stages. The results of the comparison of T2 vs. T1 summarized in Table 3 show that the PC3 score significantly distinguished T2 from T1 (*P* = 0.002). Table 4 shows the difference between TS3–TS5 compared with TS1 − TS2 and supports the elevation in PC6 and PC7 and a decrease in PC2 scores with TS3+ as summarized in Table 2. Other supporting results are given in Additional file 2: Table S1B, Additional file 1: Figures S3B and S4B. This decrease in PC2 was also seen in the comparison of TS4–TS5 vs. TS1–TS3 (Table 5). However, the other patterns observed in Table 2 with an increase in PC4 and PC5 and a decrease in PC1 scores were not seen in the logistic regression models.

**Table 3:** Multinomial logistic regression results for TS2 versus TS1 breast stage prediction

	Univariate				Multivariate			
Model	*P* value	OR	CI (95%)		*P* value	OR	CI (95%)	
Age	0.5904	1.23	0.58	2.62	0.9826	0.99	0.38	2.59
BMI	0.2044	1.22	0.90	1.66	0.6659	0.92	0.61	1.37
BCRS	0.0783	2.90	0.89	9.50				
PC1	0.4766	0.77	0.38	1.57				
PC2	0.8733	0.91	0.27	3.01	0.1928	0.25	0.03	2.00
PC3	0.0017	5.47	1.89	15.79	0.0019	12.64	2.55	62.67
PC4	0.6967	0.84	0.35	2.01				
PC5	0.9609	0.98	0.45	2.16				
PC6	0.7464	1.16	0.47	2.87	0.2224	2.00	0.66	6.06
PC7	0.8855	1.06	0.47	2.38				
PC8	0.5474	1.24	0.62	2.49				

*TS* Tanner stage, *BMI* body mass index, *BCRS* breast cancer risk score, *PC* principal component

**Table 4:** Binary logistic regression results for late (TS3–TS5) vs. early (TS1–TS2) breast stage prediction

	Univariate				Multivariate			
Model	*P* value	OR	CI (95%)		*P* value	OR	CI (95%)	
Age	<0.0001	3.32	2.03	5.42	0.1766	1.52	0.83	2.77
BMI	<0.0001	1.66	1.31	2.10	0.0020	1.59	1.19	2.14
BCRS	0.3858	0.74	0.37	1.46				
PC1	0.7848	0.94	0.61	1.45				
PC2	<0.0001	0.12	0.05	0.29	0.0011	0.08	0.02	0.37
PC3	0.0513	1.78	1.00	3.17				
PC4	0.7290	1.11	0.63	1.96				
PC5	0.2866	1.32	0.79	2.20				
PC6	0.0027	2.42	1.36	4.32	0.0371	2.47	1.06	5.67
PC7	0.0078	2.25	1.24	4.09	0.0252	2.74	1.13	6.64
PC8	0.5248	1.17	0.72	1.91				

*TS* Tanner stage, *BMI* body mass index, *BCRS* breast cancer risk score, *PC* principal component

**Table 5:** Binary logistic regression results for TS4–TS5 vs. TS1–TS3 breast stage prediction

	Univariate				Multivariate			
Model	*P* value	OR	CI (95%)		*P* value	OR	CI (95%)	
Age	<0.0001	2.65	1.75	4.01	0.0129	1.86	1.14	3.04
BMI	0.0002	1.54	1.23	1.93	0.0011	1.55	1.19	2.01
BCRS	0.9185	0.96	0.43	2.14				
PC1	0.4113	0.81	0.49	1.34				
PC2	0.0012	0.23	0.09	0.55	0.0474	0.26	0.07	0.99
PC3	0.3811	1.34	0.69	2.61				
PC4	0.4040	1.33	0.68	2.63				
PC5	0.2123	1.46	0.81	2.65				
PC6	0.3430	1.31	0.75	2.31				
PC7	0.1585	1.58	0.84	3.00				
PC8	0.2050	1.52	0.80	2.92				

*TS* Tanner stage, *BMI* body mass index, *BCRS* breast cancer risk score, *PC* principal component

**Fig. 1:**
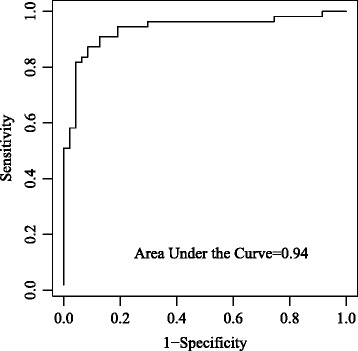
Accuracy of the predictor determined by receiver operator characteristic curve

In the LDA analysis to examine the predictive power of the model (Table 4, multivariate analysis) of OS PC in classifying girls into early stage (TS1–TS2) or late stage (TS3–TS5), the predictors in the test set of data had a reasonable multivariate normal distribution, which was the underlying assumption in LDA analysis. In the training set of data (*n* = 62), 56/62 (90%) of girls were classified correctly by the discriminant function obtained from the predictions. The cross-validated error rate was 11%. In the test set of data (*n* = 40), the discriminant function correctly classified 34/40 (85%) of girls (data not shown). Further, a receiver operator characteristic (ROC) area under the curve (AUC) of 0.94 confirmed that the accuracy of the predictor obtained by age, BMI, PC2, PC6, and PC7 was able to distinguish the early from the late breast stage (Fig. 1).

### The association between OS measurements and BCRS

The scores of three OS components (PC4, PC5, and PC8) together best predicted BCRS, even after accounting for age and BMI. Additional file 2: Table S2A summarizes the correlation analysis and scatter plots are given in Additional file 1: Figure S5. These and simple linear regression analysis (Table 6) showed that scores PC4 and PC8 are negatively correlated with BCRS, indicating that girls in the 75th percentile of these variables tend to have a lower BCRS. Multivariate regression analysis showed that age and BMI are not associated with BCRS.

**Table 6:** Breast cancer risk score (BCRS): simple and multivariate regression results

Predictor	Regression coefficient	CI (95%)		*P* value
Simple regression analysis				
Age	0.004	−0.002	0.011	0.2001
BMI	−0.0004	−0.0035	0.0026	0.7677
PC1	−0.002	−0.012	0.009	0.7526
PC2	−0.006	−0.012	0.001	0.4779
PC3	0.004	−0.009	0.017	0.5611
PC4	−0.015	−0.028	−0.001	0.0328
PC5	0.010	−0.002	0.023	0.0907
PC6	0.002	−0.009	0.014	0.7141
PC7	−0.001	−0.013	0.012	0.8963
PC8	−0.015	−0.026	−0.004	0.0082
Multiple regression analysis (best model by AIC)				
PC4	−0.011	−0.025	0.002	0.0969
PC5	0.009	−0.003	0.020	0.1480
PC8	−0.015	−0.026	−0.004	0.0076

*BMI* body mass index, *PC* principal component, *AIC* Akaike’s information criteria

The PC5 score was slightly positively correlated with BCRS, indicating a trend toward higher PC5 scores being associated with a higher BCRS. The best model found by subset selection in multiple linear regression models (Table 6) showed that PC4, PC5, and PC8 scores together best predict BCRS. The BCRS of a girl at the 75th scores percentile of PC4 or PC8 was expected to be about 0.011 or 0.015 lower than that of a girl at the 25th percentile of PC4 or PC8 respectively. BCRS for girls with the 75th percentile of PC5 was expected to be about 0.009 higher than girls in the 25th percentile of PC5. We re-examined the association between OS measurements and BCRS in the subgroup of TS3–TS5. Correlation analysis (Additional file 2: Table S2B) and simple linear regression analysis (Table 7) showed that scores PC4 and PC8 remained negatively correlated with BCRS, indicating that those with higher scores in these variables tend to have lower BC risk. The best-fitting model (Table 7) included age, PC4, and PC8 scores. BCRS for girls in the 75th percentile of PC4 or PC8 scores are expected to be about 0.017 or 0.014 lower in BCRC than for girls in the 25th percentile, respectively.

**Table 7:** Breast cancer risk score (BCRS): simple and multivariate regression results in the late (TS3 − TS5) subgroup

Predictor	Regression coefficient	CI (95%)		*P* value
Simple regression analysis				
Age	0.007	−0.001	0.016	0.0849
BMI	0.001	−0.003	0.004	0.7802
PC1	−0.004	−0.016	0.009	0.5450
PC2	−0.006	−0.030	0.019	0.6447
PC3	−0.0003	−0.0183	0.0177	0.9708
PC4	−0.020	−0.035	−0.006	0.0074
PC5	0.010	−0.005	0.025	0.1697
PC6	−0.005	−0.021	0.010	0.5014
PC7	0.007	−0.010	0.024	0.4013
PC8	−0.021	−0.037	−0.005	0.0114
Multiple regression analysis (best model by AIC)				
Age	0.007	−0.001	0.015	0.0802
PC4	−0.017	−0.032	−0.003	0.0211
PC8	−0.014	−0.030	0.002	0.0889

*TS* Tanner stage, *BMI* body mass index, *BCRS* breast cancer risk score, *PC* principal component, *AIC* Akaike’s information criteria

## Discussion

Using PCA of visible and near-infra-red (NIR) spectra from breast tissue, we were able to capture over 99% of the variation in breast tissue optical properties through eight PCs. Unlike the linear increase with age and BMI, OS components had distinct patterns by TS suggesting that OS can be used to objectively identify breast TS.

During early-stage breast development, the majority of the optical information pertains to the skin, subcutaneous tissue including the adipose tissue and the pectoral muscle, whereas for the later TS the optical signal of the pectoral muscle is replaced by the actual breast tissue. The PC scores that are correlated with each stage are sufficient to capture the changing ratios of muscle to adipose to glandular tissue within the optically sampled volume in girls’ chests during puberty.

Spectroscopically, the most striking features in the PC spectra are the strong peaks at 930 nm and 970 nm representing lipid and water absorption, respectively. These peaks both appear inversely in PC1 and are visible in PC2, PC3, PC5, and PC6, and are not statistically significant, reflecting a change in the adipose (lipid) and proliferating glandular (water) tissue. While the spectral components of the main tissue chromophores are overlapping (see Additional file 1: Figure S1C), the short wavelength range is dominated by the hemoglobins, whereas the long wavelength range is affected by collagen [26].

The current PCA analysis, while being somewhat difficult to visualize, nevertheless provides strong evidence of the ability to stage breast development in an objective manner. Each of the current PCs carries information on the various tissue chromophores as shown in Additional file 2: Table S3. The final separation of the chromophores requires significant additional computation. As Additional file 2: Table S3 illustrates, the separate PCs are related to a set of chromophores but it is the direction of these relationships and the strengths of these associations that change as the breast develops. In Additional file 2: Table S3, we show the correlation and the *P* values for PC1–8 and each chromophore. PC1, which accounts for the greatest variation, is dominated by the overall attenuation rather than the contributions of specific chromophores. The other components, however, reveal how there is additional adipose and dense tissue as the breast develops, that the ratio between the two changes, and that there is less signal from the pectoral muscle.

For example, PC2 scores are related to the amount of dense tissue which increases as the breast matures from TS2 to TS4. For transition from TS1 to TS2, which is the onset of breast development, PC3 scores become positive and remain positive through TS4, signaling an increase in lipids or adipose tissue as the breast develops. Thus, the onset of breast development is marked by an increase in adipose tissue. In addition, the PC3 scores have a large negative component at shorter wavelengths, indicating a reduction in hemoglobin and/or myoglobin within the optical measured tissue volume, indicating breast tissue with lower relative blood volume and less contribution from the pectoral muscle compared to TS1 (see Additional file 2: Table S3). The increased relative absorption by lipids at the expense of water and hence glandular tissue is also present, as shown by the declining contribution of the PC2 scores. Transition to T3 was also marked by an increase in PC6 scores, reflecting additional lipid content and an increase in PC7 scores reflecting lower collagen.

Interestingly, although PC4 and PC5 scores did not map clearly to TS they were different by BCRS. As Additional file 2: Table S3 reveals, high PC4 scores indicate increased collagen in the optically measured tissue volume and decreased hemoglobin content and oxygenation and high PC5 scores indicate less lipid.

We identified OS-derived principal components (PC2, PC3, PC6, and PC 7) that mapped to breast developmental stage. In particular, the complementarity of spectral features in PC2 and PC6 and the unique short wavelength absorption in PC3 are sufficient to capture the changing ratio of muscle to adipose to glandular tissue in girls’ chests during puberty, as noted by the multivariate regression results (Tables 3, 4, and 5) and the variable importance random forest plots (Additional file 1: Figures S4A-B). Thus, this preliminary study suggests that OS-derived measures have the potential to predict breast developmental stage in preteen and teen girls.

Furthermore, three OS-derived principal components (PC4, PC5, and PC8 scores) together best predicted BCRS. The PC4 and PC8 scores correlated negatively and significantly with BCRS indicating that those with higher scores in these variables tend to come from BCFH- families. The PC5 scores positively correlated with BCRS implying that those with higher scores in these variables tend to come from BCFH+ families. It is of interest that the lipid-water ratio, previously identified as a breast cancer risk factor in adult women is not prominent in these spectra, but there is strong absorption at the short wavelengths and long wavelengths beyond 970 nm; this suggests that the relative hemoglobin and collagen contributions may play a role in BCFH status.

## Conclusions

We have found that a non-invasive imaging method can be used to accurately classify girls by breast developmental stage. As the onset of breast development and the duration in each stage may map to increased breast cancer susceptibility, studies of pubertal development can use objective OS imaging methods, either alone or in combination with more subjective measures of breast development based on maternal or self-report of breast development stages, to more accurately predict breast development changes over time.

## Additional files

Additional file 1: Figure S1A. OS instrument with an image of the source and detector on a forearm. **Figure S1B.** Template allowing source detector placement. **Figure S1C.** Absorption spectra provided by the five dominant absorbers in breast tissue, collagen (*black solid line*), lipid (*black dashed line*), water (*black dotted line*), hemoglobin (*gray solid line*) and oxyhemoglobin (*gray dotted line*). **Figure S2A.** PC1 (*black solid line*), PC2 (*blue dashed line*), PC3 (*red dashed-dotted line*) and PC4 (*green dotted line*). **Figure S2B.** PC5 (*black solid line*), PC6 (*blue dashed line*), PC7 (*red dashed-dotted line*) and PC8 (*green dotted line*). **Figure S3A.** Predictors of TS1–TS4. **Figure S3B.** Predictors of early (TS1–TS2) and late (TS3–TS5) pubertal stages. **Figure S4A.** Random forest feature selection for a classifier of TS1–TS4. **Figure S4B.** Random forest feature selection for a classifier of early (TS1–TS2) and late (TS3–TS5). **Figure S5.** BCRS against predictor variables. (PDF 6282 kb)
Additional file 2: Table S1A. Summary statistics for predictor variables in the breast TS groups. **Table S1B.** Summary statistics for predictor variables in the early breast development stage (TS1–TS2) and late breast development stage (TS3–TS5) groups. **Table S2A.** Correlation analysis in the full study group. **Table S2B.** Correlation analysis in the late stage (TS3–TS5) subgroup. **Table S3A.** Values of *R* for correlation between the PC spectra and chromophore spectra. **Table S3B.** Strength of PC spectra to chromophore spectra correlation as given by the *P* value. (PDF 637 kb)

## Abbreviations

AIC: Akaike’s information criteria
ANOVA: Analysis of variance
BC: Breast cancer
BCFH: breast cancer family history
BCRS: Breast cancer risk score
BMI: Body mass index
CI: Confidence interval
LDA: Linear discriminant analysis
MBD: Mammographic breast density
OR: Odds ratio
OS: Optical spectroscopy
PC: Principal component
PCA: Principal component analysis
TS: Tanner stage

## References

[1] Johnson RH, Chien FL, Bleyer A (2013). Incidence of breast cancer with distant involvement among women in the United States, 1976 to 2009. JAMA.

[2] Torre LA (2016). Global cancer incidence and mortality rates and trends–an update. Cancer Epidemiol Biomarkers Prev.

[3] Colditz GA, Rosner BA, Speizer FE (1996). Risk factors for breast cancer according to family history of breast cancer. For the Nurses’ Health Study Research Group. J Natl Cancer Inst.

[4] Biro FM, Greenspan LC, Galvez MP (2012). Puberty in girls of the 21st century. J Pediatr Adolesc Gynecol.

[5] Euling SY (2008). Examination of US puberty-timing data from 1940 to 1994 for secular trends: panel finding. Pediatrics.

[6] de Muinich Keizer SM, Mul D (2001). Trends in pubertal development in Europe. Hum Reprod Update.

[7] Bodicoat DH (2014). Timing of pubertal stages and breast cancer risk: the Breakthrough Generations Study. Breast Cancer Res.

[8] Morris NM, Udry JR (1980). Validation of a self-administered instrument to assess stage of adolescent development. J Youth Adolesc.

[9] Terry MB, et al. Comparison of clinical, maternal, and self pubertal assessments: implications for health studies. Pediatrics. 2016;138(1):e20154571. doi:10.1542/peds.2015-4571. 10.1542/peds.2015-4571 PMC4925080 27279647

[10] Boyd NF (2007). Mammographic density and the risk and detection of breast cancer. N Engl J Med.

[11] Byrne C (1995). Mammographic features and breast cancer risk: effects with time, age, and menopause status. J Natl Cancer Inst.

[12] Nelson HD (2012). Risk factors for breast cancer for women aged 40 to 49 years: a systematic review and meta-analysis. Ann Intern Med.

[13] Blackmore KM, Knight JA, Lilge L (2008). Association between transillumination breast spectroscopy and quantitative mammographic features of the breast. Cancer Epidemiol Biomarkers Prev.

[14] Blyschak KSM, Jong R, Lilge L (2004). Classification of breast tissue density by optical transillumination spectroscopy: optical and physiological effects governing predictive value. Med Phys.

[15] Blackmore KM, Knight JA, Walter J, Lilge L (2015). The association between breast tissue optical content and mammographic density in pre- and post-menopausal women. PLoS One.

[16] Knight JA (2010). Optical spectroscopy of the breast in premenopausal women reveals tissue variation with changes in age and parity. Med Phys.

[17] John EM (2016). The LEGACY girls study: growth and development in the context of breast cancer family history. Epidemiology.

[18] Cerussi A (2006). In vivo absorption, scattering, and physiologic properties of 58 malignant breast tumors determined by broadband diffuse optical spectroscopy. J Biomed Opt.

[19] Shah N (2001). Noninvasive functional optical spectroscopy of human breast tissue. Proc Natl Acad Sci U S A.

[20] Dick SL, Lilge L (2006). Optical reflectance spectroscopy for prospective studies on breast cancer risk in adolescent girls. Am J Epidemiol.

[21] Simick MK (2004). Non-ionizing near-infrared radiation transillumination spectroscopy for breast tissue density and assessment of breast cancer risk. J Biomed Opt.

[22] Antoniou AC (2004). The BOADICEA model of genetic susceptibility to breast and ovarian cancer. Br J Cancer.

[23] Antoniou AC (2008). The BOADICEA model of genetic susceptibility to breast and ovarian cancers: updates and extensions. Br J Cancer.

[24] Lee AJ (2014). BOADICEA breast cancer risk prediction model: updates to cancer incidences, tumour pathology and web interface. Br J Cancer.

[25] Akaike H (1974). A new look at the statistical model identification. IEEE Trans Autom Control.

[26] Taroni P (2009). Seven-wavelength time-resolved optical mammography extending beyond 1000 nm for breast collagen quantification. Opt Express.

