# Supplementary figures and images for: Non-invasive optical spectroscopic monitoring of breast development during puberty
Source: Breast Cancer Res. 2017 Feb 6;19:12. doi: 10.1186/s13058-017-0805-x (PMC5294901; doi:10.1186/s13058-017-0805-x)

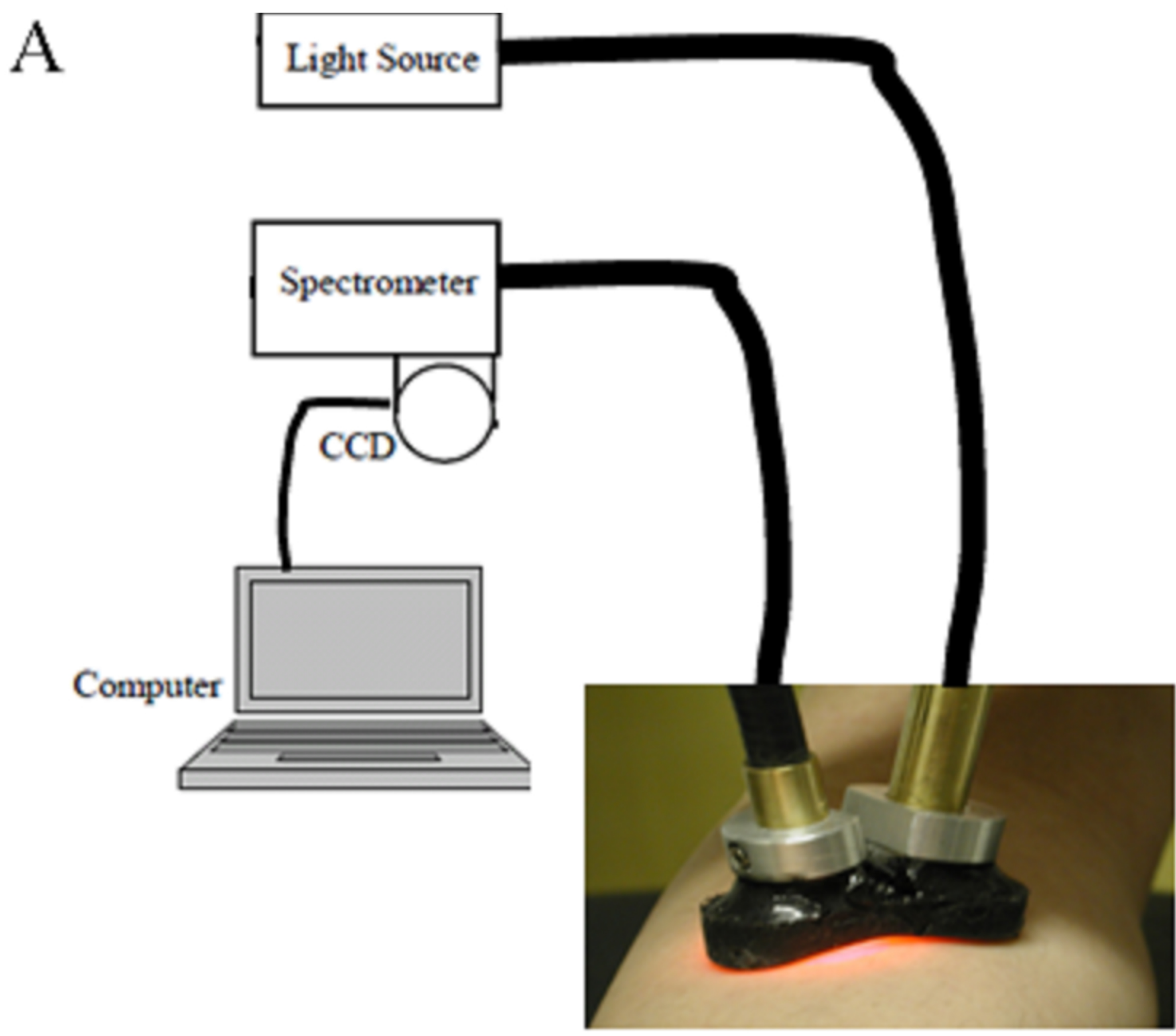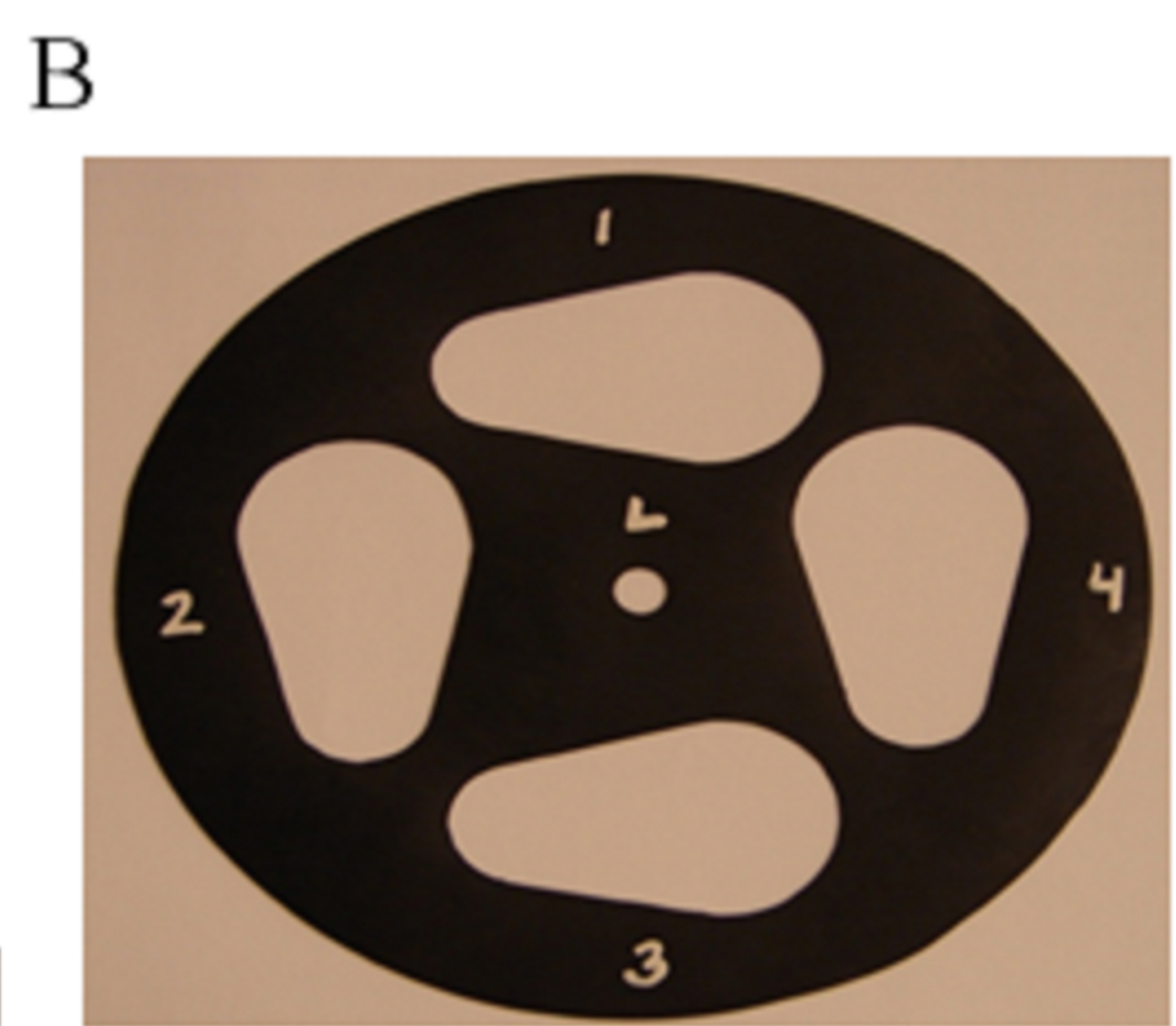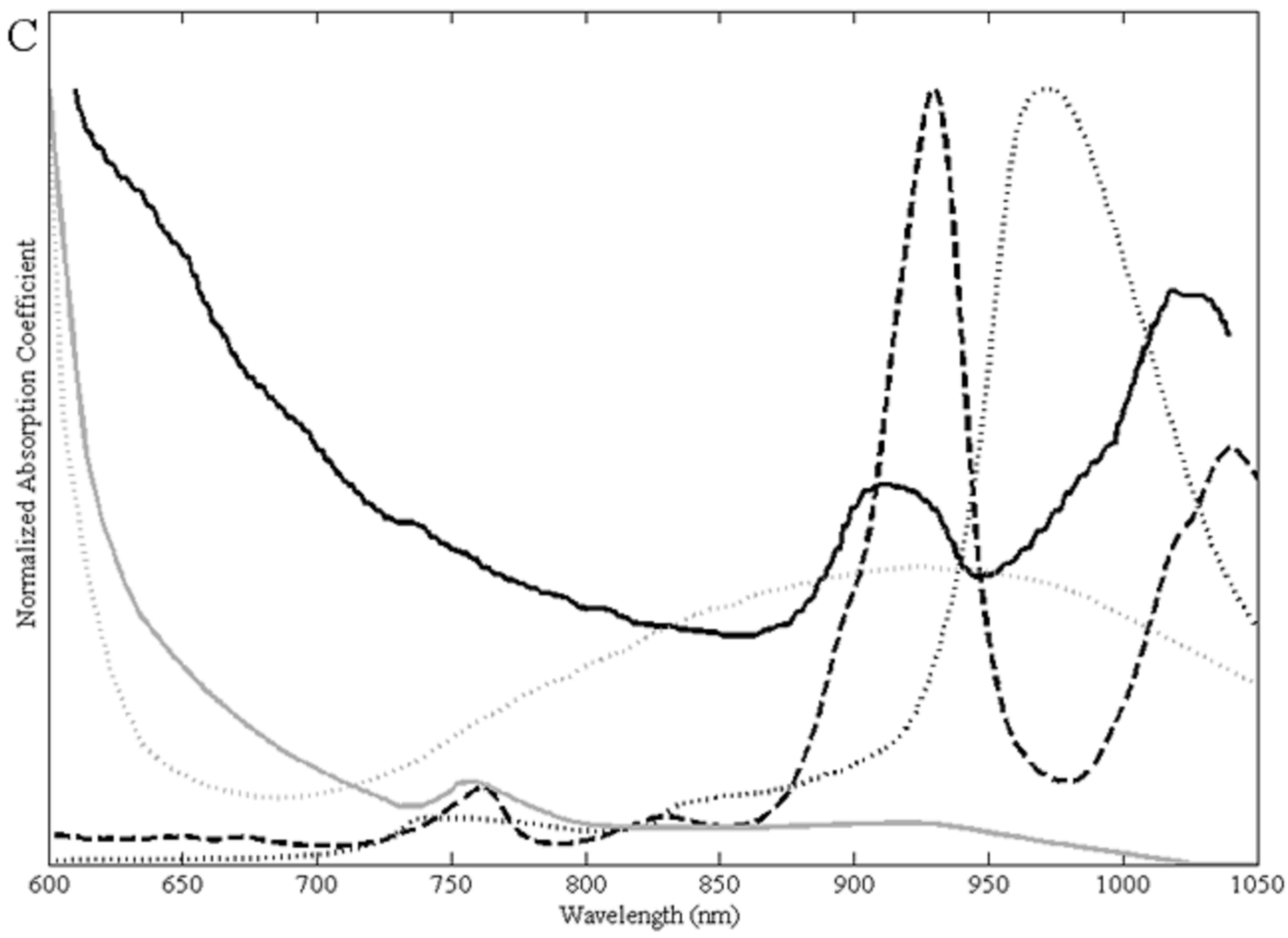

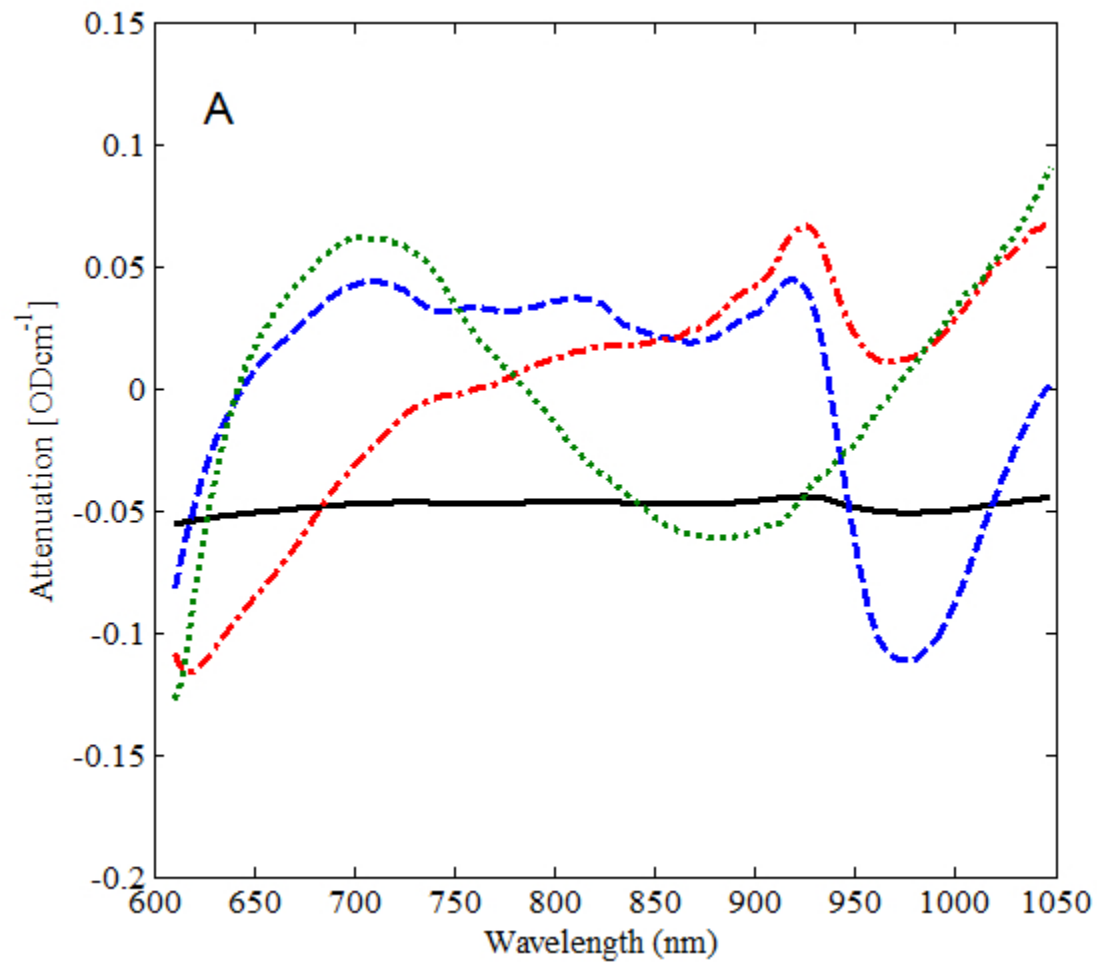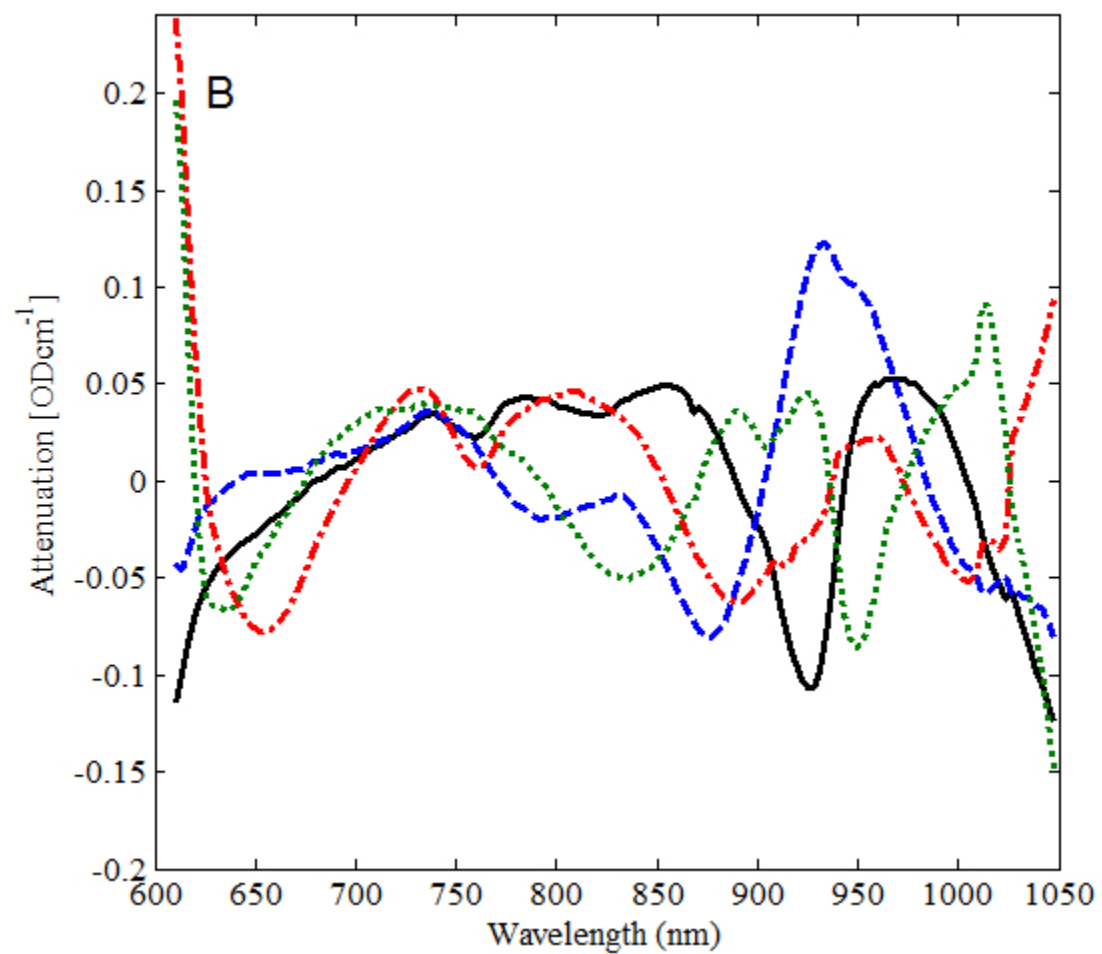

Figure S3A

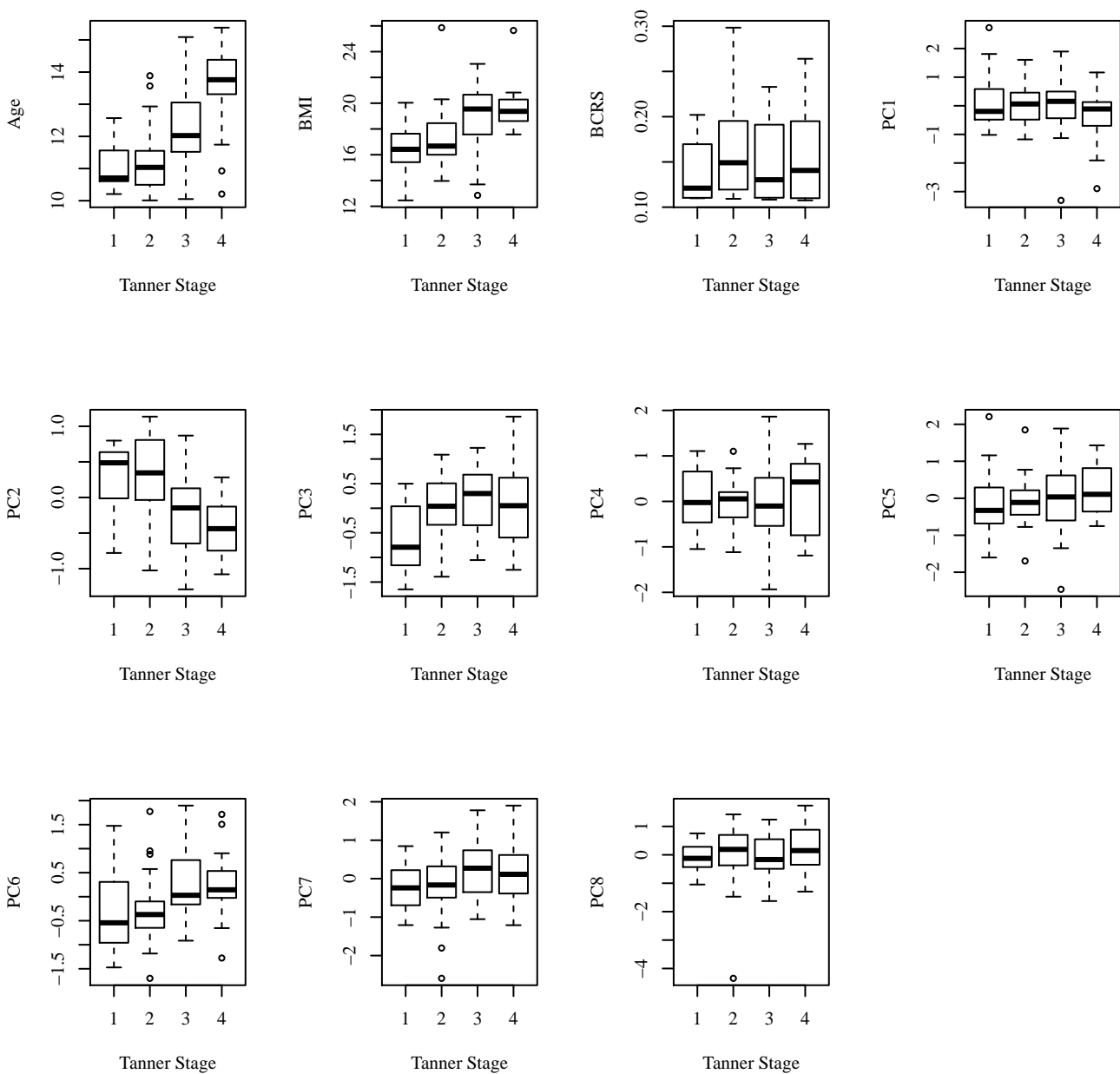

Figure S3B

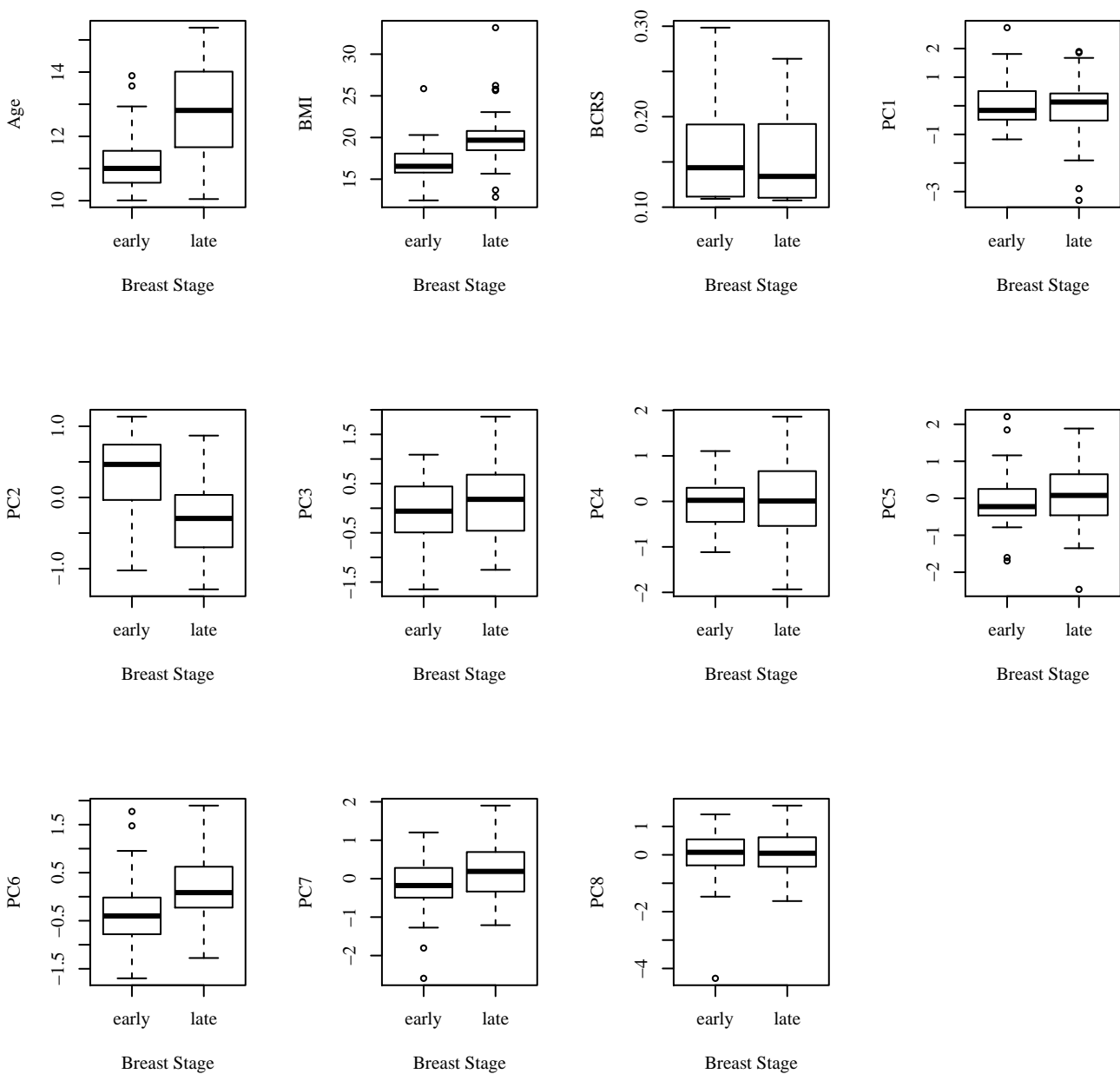

Figure S4A

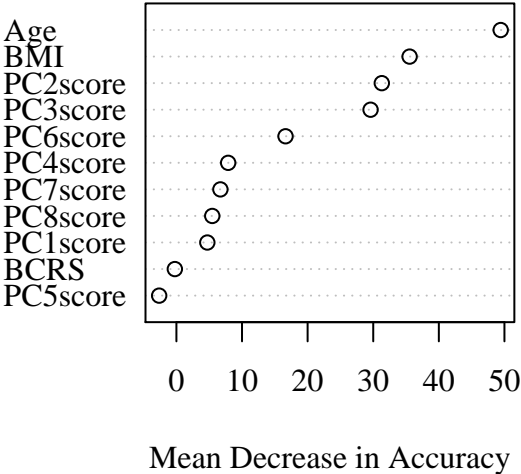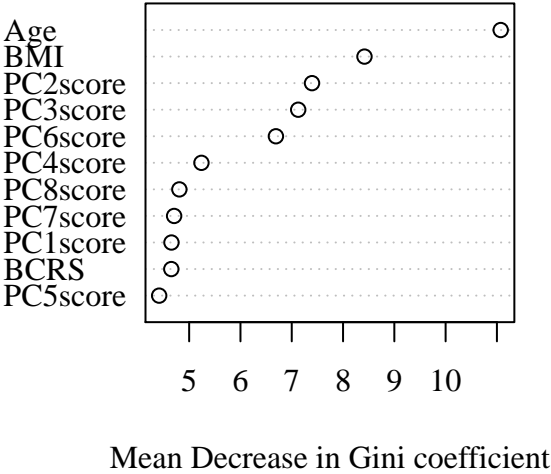

Figure S4B

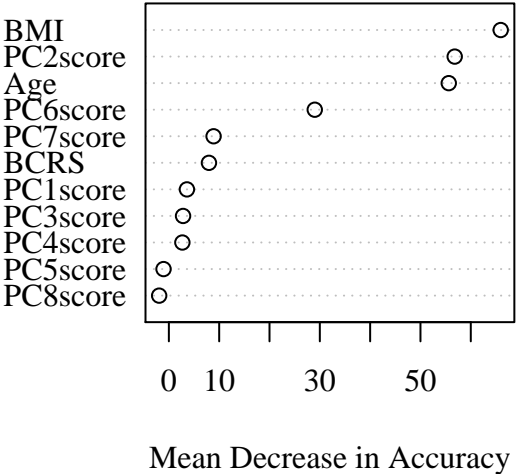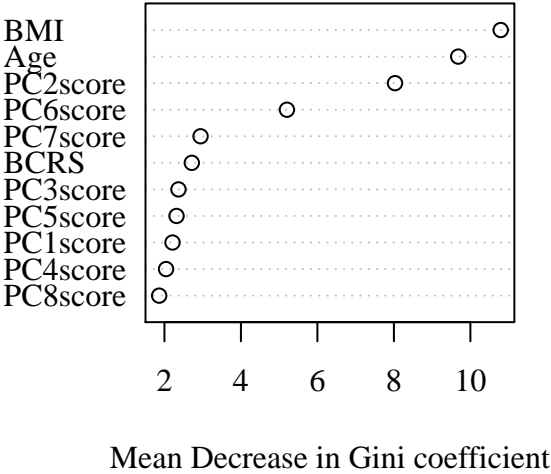

Figure S5

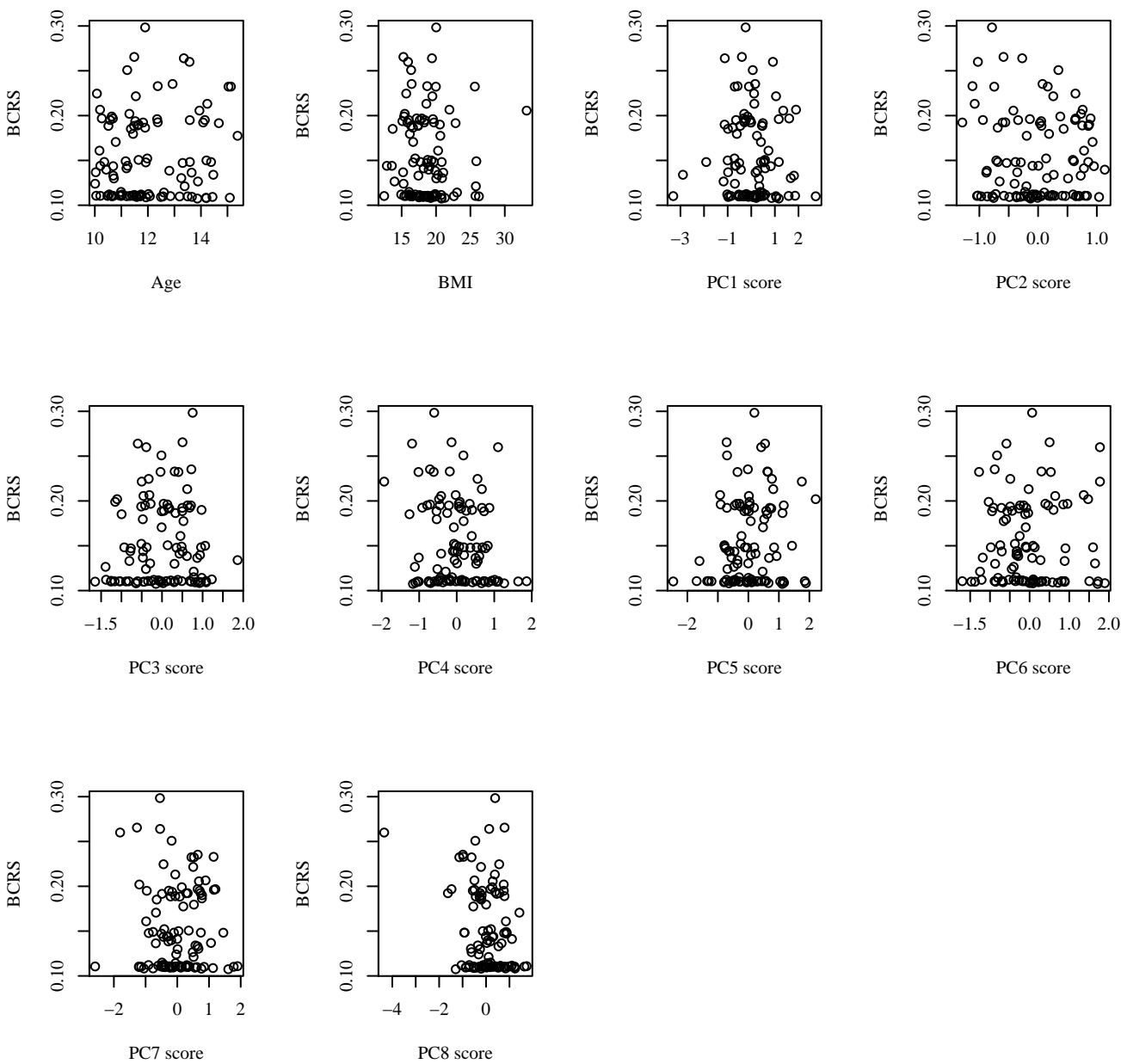

Supplement: Additional file 1: Figure S1A. — OS instrument with an image of the source and detector on a forearm. Figure S1B. Template allowing source detector placement. Figure S1C. Absorption spectra provided by the five dominant absorbers in breast tissue, collagen (black solid line), lipid (black dashed line), water (black dotted line), hemoglobin (gray solid line) and oxyhemoglobin (gray dotted line). Figure S2A. PC1 (black solid line), PC2 (blue dashed line), PC3 (red dashed-dotted line) and PC4 (green dotted line). Figure S2B. PC5 (black solid line), PC6 (blue dashed line), PC7 (red dashed-dotted line) and PC8 (green dotted line). Figure S3A. Predictors of TS1–TS4. Figure S3B. Predictors of early (TS1–TS2) and late (TS3–TS5) pubertal stages. Figure S4A. Random forest feature selection for a classifier of TS1–TS4. Figure S4B. Random forest feature selection for a classifier of early (TS1–TS2) and late (TS3–TS5). Figure S5. BCRS against predictor variables. (PDF 6282 kb) [file 13058_2017_805_MOESM1_ESM.pdf]
