# Supplementary material for: Non-invasive optical spectroscopic monitoring of breast development during puberty
Source: Breast Cancer Res. 2017 Feb 6;19:12. doi: 10.1186/s13058-017-0805-x (PMC5294901; doi:10.1186/s13058-017-0805-x)
Supplement: Additional file 2: Table S1A. — Summary statistics for predictor variables in the breast TS groups. Table S1B. Summary statistics for predictor variables in the early breast development stage (TS1–TS2) and late breast development stage (TS3–TS5) groups. Table S2A. Correlation analysis in the full study group. Table S2B. Correlation analysis in the late stage (TS3–TS5) subgroup. Table S3A. Values of R for correlation between the PC spectra and chromophore spectra. Table S3B. Strength of PC spectra to chromophore spectra correlation as given by the P value. (PDF 637 kb) [file 13058_2017_805_MOESM2_ESM.pdf]

Table S1A: Summary Statistics of predictor variables for the breast Tanner stage groups

		**Age**		**BMI**		**Breast cancer risk***	
Breast_Stage	sample_size	Mean	SD	Mean	SD	Mean	SD
1	16	11.05	0.64	16.50	1.90	1.70	0.45
2	31	11.19	0.95	17.31	2.25	2.04	0.66
3	31	12.32	1.23	18.90	2.43	1.80	0.51
4	18	13.58	1.38	19.70	1.74	1.88	0.64
		**PC1***		**PC2***		**PC3***	
Breast_Stage	sample_size	Mean	SD	Mean	SD	Mean	SD
1	16	0.19	1.01	0.33	0.43	-0.62	0.69
2	31	0.001	0.649	0.31	0.60	0.11	0.59
3	31	0.10	1.02	-0.23	0.57	0.15	0.65
4	18	-0.37	0.95	-0.41	0.42	0.10	0.80
		**PC4***		**PC5***		**PC6***	
Breast_Stage	sample_size	Mean	SD	Mean	SD	Mean	SD
1	16	0.04	0.68	-0.07	0.92	-0.34	0.88
2	31	-0.04	0.48	-0.08	0.62	-0.27	0.69
3	31	-0.03	0.82	0.01	0.92	0.28	0.82
4	18	0.14	0.86	0.22	0.66	0.24	0.72
		**PC7***		**PC8***			
Breast_Stage	sample_size	Mean	SD	Mean	SD		
1	16	-0.23	0.64	-0.12	0.51		
2	31	-0.20	0.78	0.05	1.06		
3	31	0.20	0.70	-0.01	0.67		
4	18	0.24	0.85	0.25	0.88		

*scaled by IQR (Inter-quartile range)

Table S1B: Summary Statistics of predictor variables for the early and late breast stage groups

		**Age**		**BMI**		**Breast cancer risk***	
Breast_Stage	sample_size	Mean	SD	Mean	SD	Mean	SD
early	47	11.14	0.85	17.04	2.15	1.92	0.61
late	55	12.79	1.41	19.90	3.20	1.83	0.55
		**PC1***		**PC2***		**PC3***	
Breast_Stage	sample_size	Mean	SD	Mean	SD	Mean	SD
early	47	0.07	0.78	0.31	0.54	-0.14	0.71
late	55	0.02	1.00	-0.30	0.50	0.14	0.69
		**PC4***		**PC5***		**PC6***	
Breast_Stage	sample_size	Mean	SD	Mean	SD	Mean	SD
early	47	-0.01	0.55	-0.08	0.73	-0.29	0.75
late	55	0.04	0.79	0.09	0.82	0.21	0.78
		**PC7***		**PC8***			
Breast_Stage	sample_size	Mean	SD	Mean	SD		
early	47	-0.21	0.73	-0.01	0.91		
late	55	0.20	0.72	0.09	0.73		

*scaled by IQR (Inter-quartile range)

Table S2 A: Correlation Analysis (n=102)

	Breast Cancer Risk	
**Predictor**	correlation coefficient*	P-value
Age	0.13	0.2001
BMI	-0.03	0.7677
PC1	-0.04	0.7256
PC2	-0.07	0.4779
PC3	0.06	0.5611
PC4	-0.21	0.0328
PC5	0.17	0.0907
PC6	0.04	0.7141
PC7	-0.01	0.8963
PC8	-0.26	0.0082

* Spearman's

Table S2 B: Correlation Analysis in the late subgroup (n=55)

	Breast Cancer Risk	
**Predictor**	correlation coefficient*	P-value
Age	0.23	0.0849
BMI	0.04	0.7802
PC1	-0.08	0.5450
PC2	-0.06	0.6447
PC3	-0.005	0.9708
PC4	-0.36	0.0074
PC5	0.19	0.1697
PC6	-0.09	0.5014
PC7	0.12	0.4013
PC8	-0.34	0.0114

* Spearman's

Supplementary Table S3A) Correlation R between the PC spectra and chromophore spectra

R	Hb	HbO_2	Lipid	Water	Collagen
PC1	-0.71	0.07	0.46	-0.22	-0.49
PC2	-0.01	-0.42	-0.06	-0.87	-0.15
PC3	-0.90	0.58	0.63	0.41	-0.58
PC4	-0.24	-0.69	-0.08	0.14	0.44
PC5	-0.32	-0.08	-0.67	0.09	-0.64
PC6	-0.01	0.00	0.34	0.15	-0.05
PC7	0.13	0.04	-0.12	-0.09	-0.28
PC8	0.09	0.02	0.00	0.00	-0.20

Supplementary Table S3B) significance of PC spectra to chromophore spectra correlation as given by the p value

P	Hb	HbO_2	Lipid	Water	Collagen
PC1	<0.001	0.168	<0.001	<0.001	<0.001
PC2	0.847	<0.001	0.237	<0.001	0.002
PC3	<0.001	<0.001	<0.001	<0.001	<0.001
PC4	<0.001	<0.001	0.084	0.004	<0.001
PC5	<0.001	<0.001	<0.001	0.059	<0.001
PC6	0.813	<0.001	<0.001	0.002	0.293
PC7	0.005	0.422	0.011	0.048	<0.001
PC8	0.059	0.707	0.919	0.945	<0.001
